# Autonomous Vehicle State Estimation and Mapping Using Takagi–Sugeno Modeling Approach

**DOI:** 10.3390/s22093399

**Published:** 2022-04-28

**Authors:** Shivam Chaubey, Vicenç Puig

**Affiliations:** Institut de Robòtica i Informàtica Industrial (CSIC-UPC), Llorens i Artigas, 4-6, 08028 Barcelona, Spain; shivam.chaubey1006@gmail.com

**Keywords:** simultaneous localization and mapping, linear matrix inequality, Takagi–Sugeno, linear quadratic regulator, nonlinear model-predictive control, Kalman filter

## Abstract

This paper proposes an optimal approach for state estimation based on the Takagi–Sugeno (TS) Kalman filter using measurement sensors and rough pose obtained from LIDAR scan end-points matching. To obtain stable and optimal TS Kalman gain for estimator design, a linear matrix inequality (LMI) is optimized which is constructed from Lyapunov stability criteria and dual linear quadratic regulator (LQR). The technique utilizes a Takagi–Sugeno (TS) representation of the system, which allows modeling the complex nonlinear dynamics in such a way that linearization is not required for the estimator or controller design. In addition, the TS fuzzy representation is exploited to obtain a real-time Kalman gain, avoiding the expensive optimization of LMIs at every step. The estimation schema is integrated with a nonlinear model-predictive control (NMPC) that is in charge of controlling the vehicle. For the demonstration, the approach is tested in the simulation, and for practical validity, a small-scale autonomous car is used.

## 1. Introduction

To solve the issue of traffic accidents and congestion, autonomous vehicles provide a promising solution [1]. Research work on this technology has immensely progressed from perception algorithms to vehicle control algorithms. The perception stack exploits the sensor measurements to provide the vehicle state as well as environmental information. The correction of these measurements is crucial to the safety of the vehicle when navigating within the environment. On the other hand, the planner and the controller use this information to plan the motion and achieve the desired trajectory while maintaining some objectives such as speed, time to reach the destination, and comfort. For such reasons, the measurement filtering and the obtaining of unmeasured states are paramount (e.g., lateral velocity in many cases can not be measured directly, which is important for lateral control). Advanced controllers, such as, e.g., NMPC, require a full state observation, which can be achieved using a state estimator in the loop for the full-body control.

The state estimation of an autonomous vehicle involves an algorithm that fuses the raw information provided by the sensors, which is affected by noise, and the system model, which is affected by some uncertainty represented as a disturbance. The estimation algorithm considers the measurement model, vehicle model, sensor uncertainty, and model perturbation to estimate the correct states. The standard approach is based on some form of Kalman filter, originally developed by R.E Kalman [2]. A Kalman filter provides an optimal estimate in the least-square sense, assuming that the model perfectly matches with the real system such that the noises/disturbances are Gaussian and their covariances are known. The first assumption is not always true, especially in nonlinear systems with complex dynamics. A variant named extended Kalman filter (EKF) for the nonlinear systems has been developed, which provides the solution by linearizing the system and the measurement model around the current states. The linearization is based on a Taylor series expansion, rejecting higher-order terms. The linearization process does not preserve the random variable distributions of the states and the measurements as Gaussian, which means that the optimality condition is not guaranteed. For the systems with a complex nonlinear model, such as a vehicle, the assumption of a small range of operation for a linear model acquired by linearizing the nonlinear model is not valid. For all these reasons, new methods for extending Kalman filters to nonlinear systems are required.

The TS framework offers a systematic approach to obtain a multi-model system from the original mathematical model of the system, see, e.g., for more detail, [3]. The main feature of this approach is that it allows representing the model of the nonlinear system as a set of linear system models and the overall TS model of the system is achieved by the polytopic combination of these linear system models. This allows extending the LMI design procedures for control and estimation to the nonlinear systems represented in polytopic TS form. Moreover, there exists a systematic procedure for generating the TS model for the nonlinear model and approximating it in a polytopic way [4].

In this paper, the state estimation of an autonomous vehicle is developed using an approach alternative to the use of EKF. Moreover, this approach will be combined with a LIDAR scan matching algorithm that provides a rough pose estimation. For LIDAR scan matching, the real-time correlative scan matching algorithm is being exploited instead of iterative closest point (ICP) [5], which has a drawback of expensive search for point correspondences in every iteration. As LIDAR scan matching is not within the scope of this paper, more details can be found in [6,7]. The methods such as Gmapping [8], which generally require a large number of particles to obtain good results, lead to higher computational complexity compared with our approach. In comparison with the methods such as LOAM [9], our method fuses the LIDAR and other sensor information with the accurate vehicle model prediction for precise localization. The proposed method can be extended to any degree of freedom (DoF) system if the model is known and additional sensors can be integrated. In our approach, dynamic states, such as longitudinal velocity and angular velocity, will be directly measured from the sensors. Then, the rough pose obtained from the LIDAR scan matching and direct measurements from sensors will be corrected using the TS Kalman filter design. A similar work [10] was carried out to improve the approximation error by utilizing the Takagi–Sugeno model; however, this approach considers a limited and known number of landmarks and neither ensures optimality. Another work [11] utilized a linear parameter varying (LPV) system, which is analogous to TS and has shown good performance in the simulation with different applications.

To summarize, the contributions of our work are as follows:

- A TS Kalman filter is developed which does not need linearization at each step, as in the case of EKF. In addition, the estimator provides a stable and optimal solution.
- The design of the TS Kalman filter is solved offline using LMIs while online (in real-time), only the interpolation of the TS subsystems gains is required.
- A case study of a small-scale autonomous vehicle is presented to obtain the full state by correcting the raw measurement obtained from sensors and LIDAR scan end-point matching.
- The whole framework, including NMPC, is validated on a small computation board Odroid XU4. The low computational requirements of the framework, especially SLAM, make it easy to deploy on small robots.

The structure of the paper is the following: Section 2 describes the proposed approach and presents the autonomous vehicle considered as a case study. Section 4 presents the proposed TS Kalman filter for the state estimation. Section 5 presents the simulation and experimental results using the considered case study. Finally, Section 6 draws the main conclusions of the paper and proposes future research paths.

## 2. Proposed Approach

**Figure 1:**
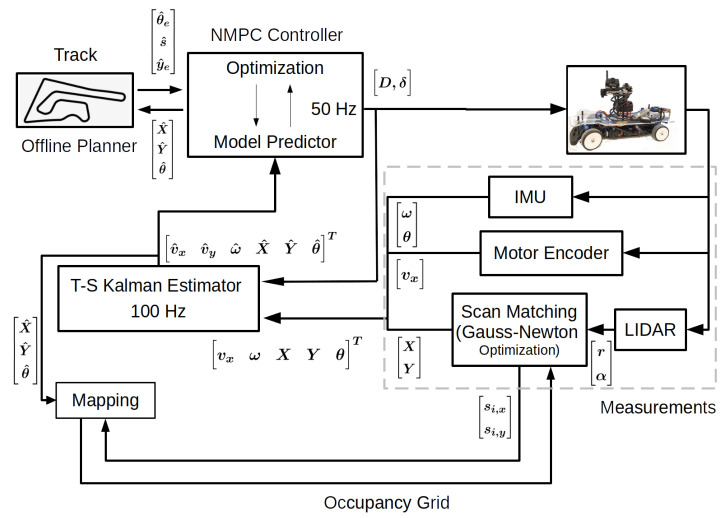
The architecture of the software modules, including information flow, and a conceptual overview of the interconnections. Software framework also includes low-level sensor measurement acquisition units.

Figure 1 presents the overall architecture and integration of different modules for the autonomy. The proposed pipeline also includes an NMPC controller. Descriptions of the states $x={\left[v_{x},v_{y},\omega ,X,Y,\theta \right]}^{T}$ and control $u={\left[D,\delta \right]}^{T}$ symbol are listed in Table 1. The NMPC controller uses the error model of the system so the estimated states ${\left[\hat{X},\hat{Y},\hat{\theta }\right]}^{T}$ are converted to the errors states ${\left[{\hat{\theta }}_{e},\hat{s},{\hat{y}}_{e}\right]}^{T}$ using the offline planner; details can be found in [12]. For the state measurements, an inertial measurement unit (IMU), a motor encoder, and scan end-point matching modules are utilized. The mapping module is dedicated to map the environment using the scanned points and estimated pose of the vehicle. The previous map (occupancy grid) is queried by the scan-matching module to roughly estimate the relative position of the current scan. Finally, the estimator module is responsible for correcting the uncertainty in the measurements and scan matching, as well as obtaining the hidden state $v_{y}$.

To generalize the SLAM algorithm, i.e., free from the number of landmarks and environment, the rough pose estimation is based on LIDAR scan end-point matching by exploiting the Gauss–Newton optimization process. This matching approach allows estimating the pose without any prior information of the environment or landmarks. The scan end-points matching is similar to the work carried out in the paper [6], which is presented in Section 3. In this development, LIDAR’s scan end-points are exploited for observing the environment. Instead, a camera can also be used for the rough localization using either feature matching technique [13] or deep learning technique [14]. Then, to correct the rough pose from the scan matching and acquired sensor measurements, a TS modeling approach is considered to design an optimal and stable Kalman estimator. The whole design procedure consists of the following steps:

1. Derive a TS model from the nonlinear model which embeds the nonlinearity term inside the system matrices.
2. Obtain the polytopic systems and derive the fuzzy representation of the TS model [4].
3. Formulate LMIs for Lyapunov stability and optimal dual LQR for all the obtained polytopes or system vertices of the fuzzy model to obtain the offline Kalman gain.
4. Exploit fuzzy interpolation technique to find the online gain for the TS Kalman filter.
5. Apply online TS Kalman gain for the state estimation.

Steps 3 to 5 are presented thoroughly in Section 4 of this paper, while the remaining steps are detailed in this section.

Formalizing the TS model for LMIs and fuzzy gain interpolation technique is based on the concept of fuzzy theory [4,15], which offers a systematic approach to obtain a multi-model system from the original mathematical model of the system. The main feature of this model is to express the local dynamics of each fuzzy implication by a set of linear system models, and the overall fuzzy model of the system is achieved by the fuzzy blending of these linear system models. It is proved that TS fuzzy models are universal approximators with a high degree of precision of any smooth nonlinear system [16].

### 2.1. Considered Autonomous Vehicle

To validate the proposed approach, a case based on a small-scale autonomous car is used. The states of the car have to be estimated using available sensors, and, further, this information will be exploited by the full-body controller.

The dynamic states, such as longitudinal velocity, can be roughly estimated using the radius and RPM of the rear wheel measured by the motor encoder. Angular velocity can be measured by an IMU sensor. The rough position of the vehicle can be obtained using the scan end-point matching algorithm and orientation from IMU. Figure 1 shows the high-level scheme of the proposed pipeline which includes a model-predictive controller (MPC) in the loop. We do not provide further details about the MPC algorithm, as this is not the focus of this work. We assume that the control of the car and the track plan can be obtained.

**Figure 2:**
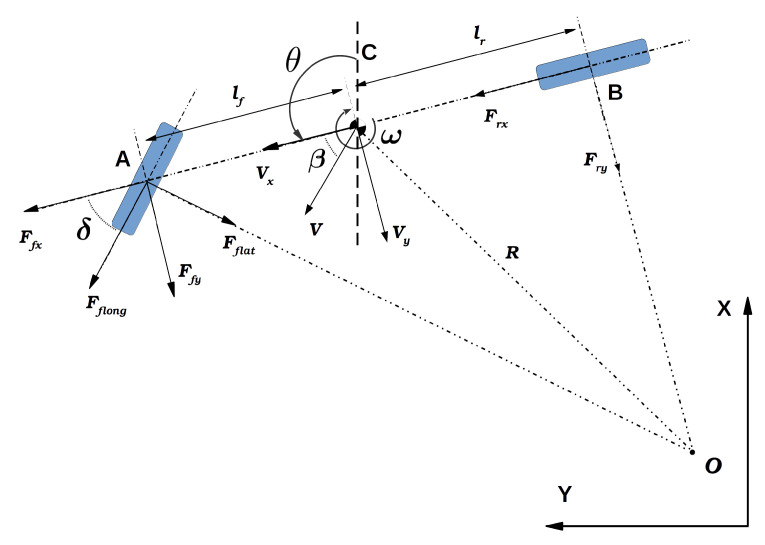
A representation of the bicycle model in 2D space for deriving the equations of motion. The vehicle schematic shows the inertial frame (O), the center of gravity (CoG) frame (C) attached to the center of gravity of the vehicle. The forces $F_{rx}$ and $F_{ry}$ on the rear wheel are the longitudinal and lateral force, respectively. The front wheel forces $F_{flong}$ and $F_{flat}$ are the longitudinal and lateral forces, respectively, the forces $F_{fx}$ and $F_{fy}$ are the result of these forces in frame C. The lengths $l_{f}$ and $l_{r}$ are the distance from CoG to the front wheel and rear wheel, respectively.

As the estimator is based on a system model, the dynamics of the system are derived using the bicycle model [17] representation shown in Figure 2. The vehicle model includes kinematic as well as dynamic equations, represented by:

(1) $$\begin{array}{cc} \dot{v_{x}} & =\frac{1}{m}\left(F_{rx}-F_{flat}\sin\left(\delta \right)+mv_{y}\omega \right)\\ \dot{v_{y}} & =\frac{1}{m}\left(F_{flat}\cos\left(\delta \right)+F_{ry}-mv_{x}\omega \right)\\ \dot{\omega } & =\frac{1}{I_{z}}\left(l_{f}F_{flat}\cos\left(\delta \right)-l_{r}F_{ry}\right)\\ \dot{X} & =v_{x}cos\left(\theta \right)-v_{y}sin\left(\theta \right)\\ \dot{Y} & =v_{x}sin\left(\theta \right)+v_{y}cos\left(\theta \right)\\ \dot{\theta } & =\omega \end{array}$$

where $v_{x},v_{y},\omega$ are the longitudinal, lateral, and angular velocity of the vehicle, respectively. X,Y,θ are the global pose of the vehicle in a fixed inertial frame. The available sensors are able to measure, directly or indirectly, $\left[v_{x},\omega ,X,Y,\theta \right]$ states, with some errors. The lateral velocity $v_{y}$ cannot be measured and will be estimated. $F_{rx}$ is the longitudinal force in the rear wheel which consists of force from the motor, drive-line resistance, and drag force. $F_{flat}$ and $F_{ry}$ are the lateral tire force in the front and rear wheel, respectively, which is obtained by simplifying the Pacejka tire model [18]. Longitudinal force on the front wheel is considered to be negligible since no brake nor torque is applied to it. The forces are given by

(2) $$F_{rx}=\left(C_{m0}-C_{m_{1}}v_{x}\right)D-C_{0}v_{x}-C_{1}-\frac{C_{D}A\rho v_{x}^{2}}{2}$$

(3) $$F_{flat}=2C_{af}\left(\delta -\arctan\left(\frac{v_{y}+l_{f}\dot{\theta }}{v_{x}}\right)\right)$$

(4) $$F_{ry}=-2C_{ar}\arctan\left(\frac{v_{y}-l_{r}\dot{\theta }}{v_{x}}\right)$$

where δ and *D* are two control inputs, steering angle and duty cycle, respectively. Some of the system parameters are obtained by physical measurement, and the remaining ones by least-squares optimization. The obtained values and description of the parameters are listed in Table 2.

#### 2.1.1. Construction of TS Model

The goal is to derive a TS model from the nonlinear vehicle dynamics (1) as if the response of the TS model exactly matches the response of a nonlinear system within a specified domain with the same input *u*. To form the TS model, the varying nonlinear terms in the equations are set as fuzzy variables or scheduling variables $\left(\vartheta_{i}\left(t\right)\right)$.

The set of fuzzy (or scheduling) variables is represented in Table 3. These are the state and control variables on which the nonlinear dynamics are dependent. These variables represent the physical limit of the vehicle, for example, the maximum and minimum longitudinal velocities or maximum and minimum steering angle that a vehicle can achieve.

From the nonlinear model (1), the TS model (5) is obtained by embedding schedule variables inside the matrix:

(5) $$\underset{\dot{x}}{\underset{︸}{\left[\begin{array}{c} \dot{v_{x}}\\ \dot{v_{y}}\\ \dot{\omega }\\ \dot{X}\\ \dot{Y}\\ \dot{\theta } \end{array}\right]}}=\underset{A(\vartheta )}{\underset{︸}{\left[\begin{array}{cccccc} A_{11} & A_{12} & 0 & 0 & 0 & 0\\ A_{21} & A_{22} & 0 & 0 & 0 & 0\\ A_{31} & 0 & 0 & 0 & 0 & 0\\ A_{41} & A_{42} & 0 & 0 & 0 & 0\\ A_{51} & A_{52} & 0 & 0 & 0 & 0\\ 0 & 0 & 1 & 0 & 0 & 0 \end{array}\right]}}\underset{x}{\underset{︸}{\left[\begin{array}{c} v_{x}\\ v_{y}\\ \omega \\ X\\ Y\\ \theta \end{array}\right]}}+\underset{B(\vartheta )}{\underset{︸}{\left[\begin{array}{cc} B_{11} & B_{12}\\ 0 & B_{22}\\ 0 & B_{32}\\ 0 & 0\\ 0 & 0\\ 0 & 0 \end{array}\right]}}\underset{u}{\underset{︸}{\left[\begin{array}{c} D\\ \delta \end{array}\right]}}$$

where $A_{i,j}$ and $B_{i,j}$ are the varying sub-elements of matrix A(ϑ) and matrix B(ϑ), respectively, and can be expressed as

(6) $$\begin{array}{c} A_{11}=-\frac{1}{m}\left(C_{0}+\frac{C_{1}}{v_{x}}+\frac{C_{D}A\rho v_{x}}{2}\right),\;A_{12}=\omega ,\\ A_{21}=-\omega ,\;A_{22}=-\frac{2C_{ar}}{mv_{y}}\arctan\left(\frac{v_{y}-l_{r}\dot{\theta }}{v_{x}}\right),\\ A_{31}=\frac{2C_{ar}l_{r}}{v_{x}I_{z}}\arctan\left(\frac{v_{y}-l_{r}\dot{\theta }}{v_{x}}\right),\\ A_{41}=cos\left(\theta \right),\;A_{42}=-sin\left(\theta \right),\\ A_{51}=sin\left(\theta \right),\;A_{52}=cos\left(\theta \right)\\ B_{11}=\frac{1}{m}\left(C_{m0}-C_{m_{1}}v_{x}\right),\\ B_{12}=-\frac{2C_{af}}{m\delta }\left(\delta -\arctan\left(\frac{v_{y}+l_{f}\dot{\theta }}{v_{x}}\right)\right)\sin\left(\delta \right)\\ B_{22}=\frac{2C_{af}}{m\delta }\left(\delta -\arctan\left(\frac{v_{y}+l_{f}\dot{\theta }}{v_{x}}\right)\right)\cos\left(\delta \right),\\ B_{32}=\frac{2C_{af}l_{f}}{I_{z}\delta }\left(\delta -\arctan\left(\frac{v_{y}+l_{f}\dot{\theta }}{v_{x}}\right)\right)\cos\left(\delta \right) \end{array}$$

The scheduling variables can be further represented as a set of membership function $\vartheta_{i}$, as follows:

(7) $$\begin{array}{c} \vartheta_{i}\left(t\right)=\mu_{i1}\left(\vartheta_{i}\left(t\right)\right)\cdot \underline{\vartheta_{i}})+\mu_{i2}\left(\vartheta_{i}\left(t\right)\right)\cdot \bar{\vartheta_{i}},\;i\in \left[1,2,\ldots ,5\right] \end{array}$$

(8) $$\begin{array}{c} \mu_{i1}\left(\vartheta_{i}\left(t\right)\right)+\mu_{i2}\left(\vartheta_{i}\left(t\right)\right)=1, \end{array}$$

where $\underline{\vartheta_{i}}$, $\bar{\vartheta_{i}}$ are the minimum and maximum scheduling variables, respectively, and the $\mu_{ij}\left(\vartheta_{i}\left(t\right)\right)$ are the fuzzy sets with two membership functions for each scheduling variable being in total 32 combinations. For each membership function, a system vertex or polytopic system represented by $A_{i}x\left(t\right)+B_{i}u\left(t\right)$ can be obtained, and by applying the fuzzy rules, a nonlinear system can be modeled as:

(9) $$\begin{array}{c} \dot{x}\left(t\right)=\sum_{i=1}^{32}h_{i}\left(\vartheta \left(t\right)\right)\left\{A_{i}x\left(t\right)+B_{i}u\left(t\right)\right\} \end{array}$$

where,

(10) $$\begin{array}{c} \vartheta \left(t\right)=[\vartheta_{1}\left(t\right),\vartheta_{2}\left(t\right),\vartheta_{3}\left(t\right),\vartheta_{4}\left(t\right),\vartheta_{5}\left(t\right)] \end{array}$$

and

(11) $$\begin{array}{c} h_{i}\left(\vartheta \left(t\right)\right)=\mu_{1j}\left(\vartheta_{1}\left(t\right)\right)\cdot \mu_{2j}\left(\vartheta_{2}\left(t\right)\right)\cdot \mu_{3j}\left(\vartheta_{3}\left(t\right)\right)\cdot \mu_{4j}\left(\vartheta_{4}\left(t\right)\right)\cdot \mu_{5j}\left(\vartheta_{5}\left(t\right)\right),\;j\in \left[1,2\right] \end{array}$$

The polytopic system representation will be used to formulate the LMIs to obtain offline gain in Section 4.1. Additionally, the fuzzy representation (9) of the TS model will also allow the computation of online gain using the interpolation technique without optimizing the LMIs at each time step.

#### 2.1.2. Measurement Model

The rough position of the vehicle is obtained from the scan matching algorithm, and other information is directly obtained from various sensors listed in Table 4.

The discretized state-space measurement model for the system is defined by $y_{k}=Cx_{k}+Du_{k}+E_{v}v_{k}$, where $v_{k}\in R^{n_{y}}$ is the measurement noise and $n_{y}=5$, $E_{v}$ is the distribution matrix with appropriate dimension. $D=0_{5\times 2}$ is taken as there is no interaction from control input. The *C* matrix relates the system state to the output measurement $y_{k}$.

(12) $$E_{v}=\left[\begin{array}{ccccc} 1 & 0 & 0 & 0 & 0\\ 0 & 1 & 0 & 0 & 0\\ 0 & 0 & 1 & 0 & 0\\ 0 & 0 & 0 & 1 & 0\\ 0 & 0 & 0 & 0 & 1 \end{array}\right],\;\;\;C=\left[\begin{array}{cccccc} 1 & 0 & 0 & 0 & 0 & 0\\ 0 & 0 & 1 & 0 & 0 & 0\\ 0 & 0 & 0 & 1 & 0 & 0\\ 0 & 0 & 0 & 0 & 1 & 0\\ 0 & 0 & 0 & 0 & 0 & 1 \end{array}\right]$$

The noise vector $v_{k}={\left[\sigma_{v_{x}}^{2},\sigma_{\omega }^{2},\sigma_{X}^{2},\sigma_{Y}^{2},\sigma_{\theta }^{2}\right]}^{T}$ has noise of each sensor with the covariance listed in Table 4.

#### 2.1.3. Observability Analysis

Before designing the estimator, the observability of Equation (5) needs to be analyzed for singularity. This equation consists of $n_{x}=6$ state variables and the observability matrix defined by:

(13) $$O={\left[\begin{array}{c} C,CA_{d},CA_{d}^{2},\cdot \cdot \cdot ,CA_{d}^{n_{x}-1} \end{array}\right]}^{T}$$

If the rank of observability is equal to $n_{x}$, then the system is observable. To check the observability, Equation (5) is discretized using Euler approximation $A_{d}=I+A\cdot \Delta t_{s}$. During the analysis, at certain states, the rank of the observability matrix O reaches singularity, precisely when any of the $v_{x}=0,v_{y}=0$ and δ=0. To resolve this issue whenever this state variable attains this value, a bias ϵ=0.0001 is added to this variable.

## 3. SLAM Algorithm

The measurement from the LIDAR provides the environment information through building an occupancy grid probability map using Bayes inference theory which is later on converted to occupancy grids for detecting obstacles and free zones within the map. Using the occupancy probability map at time {t} and previous probability maps {t−1:0}, a robust matching is performed using Gauss–Newton optimization to obtain the relative pose. This approach allows to map and estimate the pose without any prior information about the environment or landmarks. Later on, this pose and map will be cured using the TS estimator due to corrupted matching as the LIDAR has an error in the measurement. For robust matching between occupancy probability maps, the sub-grid-level probability is accessed for sub-grid-level accuracy [6]. The algorithm has two main steps: (a) mapping the obtained map into sub-grid level accuracy, and (b) localization using the optimization process.

### 3.1. Mapping

**Figure 3:**
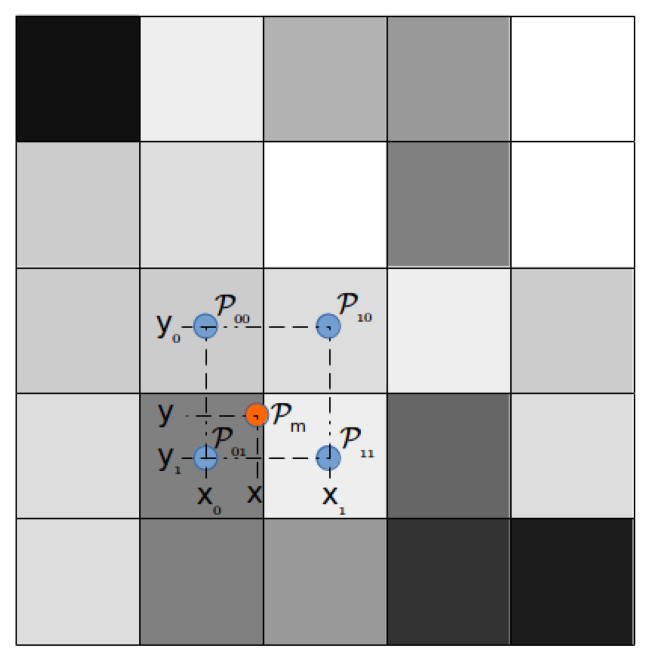
Calculation of laser end-point probability at sub-grid level by using occupancy grid probability map.

The map used for matching is developed using an interpolation scheme to overcome the discrete nature of the map through bilinear filtering, which allows sub-grid-level cell accuracy for estimating occupancy probabilities and derivatives. The occupancy value $M(P_{m})$ can be approximated by linear interpolation of a scan end-point $P_{m}$, similar to Figure 3, in a continuous map coordinate given as:

(14) $$\begin{array}{c} M\left(P_{m}\right)\approx \frac{Y-Y_{0}}{Y_{1}-Y_{0}}\left(\frac{X-X_{0}}{X_{1}-X_{0}}M\left(P_{11}\right)+\frac{X_{1}-X}{X_{1}-X_{0}}M\left(P_{01}\right)\right)\\ +\frac{Y_{1}-Y}{Y_{1}-Y_{0}}\left(\frac{X-X_{0}}{X_{1}-X_{0}}M\left(P_{10}\right)+\frac{X_{1}-X}{X_{1}-X_{0}}M\left(P_{00}\right)\right) \end{array}$$

where $P_{00},P_{01},P_{10}$, and $P_{11}$ are the closest integer point coordinates in a grid map. The partial derivatives along the *X* and *Y* axis can be approximated by:

(15) $$\begin{array}{c} \frac{\partial M}{\partial X}\left(P_{m}\right)\approx \frac{Y-Y_{0}}{Y_{1}-Y_{0}}\left(M\left(P_{11}\right)-M\left(P_{01}\right)\right)\\ +\frac{Y_{1}-Y}{Y_{1}-Y_{0}}\left(M\left(P_{10}\right)-M\left(P_{00}\right)\right)\\ \frac{\partial M}{\partial Y}\left(P_{m}\right)\approx \frac{X-X_{0}}{X_{1}-X_{0}}\left(M\left(P_{11}\right)-M\left(P_{10}\right)\right)\\ +\frac{X_{1}-X}{X_{1}-X_{0}}\left(M\left(P_{01}\right)-M\left(P_{00}\right)\right) \end{array}$$

### 3.2. Localization

**Figure 4:**
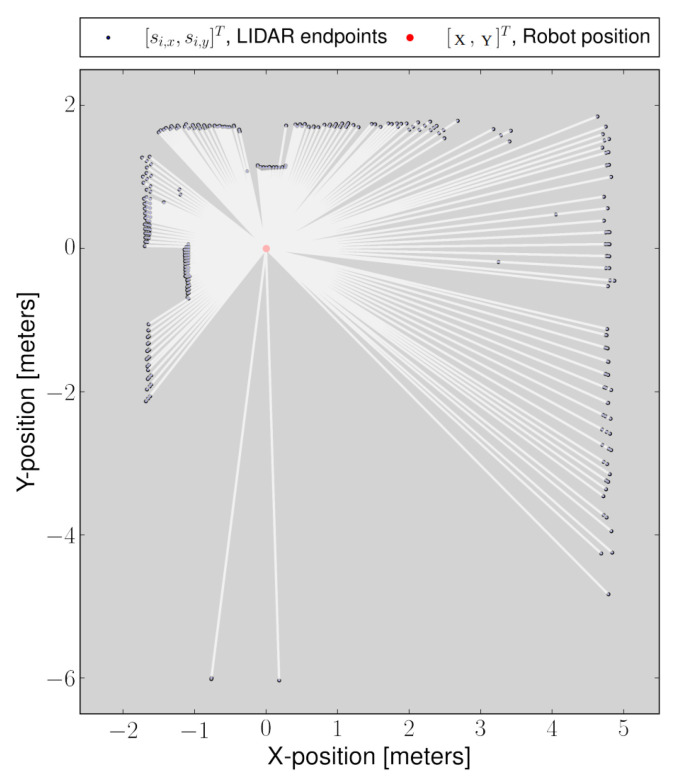
Translation of scan information ${\left[r,\alpha \right]}^{T}$ to scan end-points ${\left[s_{iX},s_{iY}\right]}^{T}$ in LIDAR frame of reference.

The localization algorithm is based on the matching of scan end-points shown in Figure 4 with the existing maps to find the rigid transformation $\xi ={\left(X,Y,\theta \right)}^{T}$ that minimizes

(16) $$\xi^{*}=\arg\min_{\xi }\sum_{i=1}^{n}{\left[1-M\left(S_{i}\left(\xi \right)\right)\right]}^{2}$$

where $S_{i}\left(\xi \right)$ is the location of scan end-points in the world coordinate frame, given by:

(17) $$S_{i}\left(\xi \right)=\left[\begin{array}{cc} cos(\theta ) & -sin(\theta )\\ sin(\theta ) & cos(\theta ) \end{array}\right]\left[\begin{array}{c} s_{i,x}\\ s_{i,y} \end{array}\right]+\left[\begin{array}{c} X\\ Y \end{array}\right]$$

where $s_{i}={\left(s_{i,x},s_{i,y}\right)}^{T}$ is the location of end-points in the LIDAR coordinate frame. The X,Y,andθ are the pose of the LIDAR in the world coordinate frame. The function $M(S_{i}\left(\xi \right))$ provides the map value at the coordinate given by $S_{i}\left(\xi \right)$. Let us suppose the robot started at initial pose ξ and control input is given to move by Δξ. At position ξ, the map is built from the measurement, and at ξ+Δξ, a new map is registered. To estimate the Δξ, the optimizer minimizes the error according to:

(18) $$\sum_{i=1}^{n}{\left[1-M\left(S_{i}\left(\xi +\Delta \xi \right)\right)\right]}^{2}\to 0$$

Solving for Δξ, using first-order Taylor expansion of $M(S_{i}\left(\xi +\Delta \xi \right))$ and setting the partial derivative with respect to ξ, yields the Gauss–Newton minimization equation:

(19) $$\Delta \xi =H^{-1}\sum_{i=1}^{n}{\left[\Delta M\left(S_{i}\left(\xi \right)\right)\frac{\partial S_{i}\left(\xi \right)}{\partial \xi }\right]}^{T}\left[1-M\left(S_{i}\left(\xi \right)\right)\right]$$

where

(20) $$H={\left[\Delta M\left(S_{i}\left(\xi \right)\right)\frac{\partial S_{i}\left(\xi \right)}{\partial \xi }\right]}^{T}\left[\Delta M\left(S_{i}\left(\xi \right)\right)\frac{\partial S_{i}\left(\xi \right)}{\partial \xi }\right]$$

and from Equation (17), the gradient of $\frac{\partial S_{i}\left(\xi \right)}{\partial \xi }$ can be represented as

(21) $$\frac{\partial S_{i}\left(\xi \right)}{\partial \xi }=\left[\begin{array}{ccc} 1 & 0 & -\sin\left(\theta \right)s_{i,x}-\cos\left(\theta \right)s_{i,y}\\ 0 & 1 & \cos\left(\theta \right)s_{i,x}-\sin\left(\theta \right)s_{i,y} \end{array}\right]$$

and the map gradient $\Delta M(S_{i}\left(\xi \right))$ can be approximated from Equation (15). The obtained pose might be inaccurate due to sensor noises and errors which need to be corrected before building the map. The estimator which is discussed in the next section will be used to correct this pose. The corrected pose is applied to Equation (17) to relocate the scan end-points $S_{i}$, which eventually updates the occupancy probability grid map.

## 4. Takagi–Sugeno Kalman Filter Design

For the development of the proposed estimator design, first, in Section 4.1, LMIs for the TS Kalman filter design are formulated. Second, in Section 4.2, the measurement noise and system perturbation matrix are provided. Finally, the implementation of the proposed approach and its improvement are discussed in Section 4.3.

### 4.1. LMI Design Procedure

The following Kalman filter to obtain the $\hat{x}$ estimation is required:

(22) $$\begin{array}{c} \dot{\hat{x}}\left(t\right)=\left(A\left(\vartheta \right)-L\left(\vartheta \right)C\right)\hat{x}+\left(B\left(\vartheta \right)-L\left(\vartheta \right)D\right)u+L\left(\vartheta \right)y \end{array}$$

where A(ϑ),B(ϑ) can be obtained by using TS model (5), L(ϑ) is the online Kalman gain, and $D=0_{5\times 2}$. The above continuous Kalman estimator is discretized for implementation on a real-time system. Now, the aim is to find the optimal L(ϑ) which converges to the estimation ground truth in the presence of sensor noise and system disturbance. To obtain an optimal Kalman gain L(ϑ), first, LMIs are offline optimized to obtain gain $L^{off}$. Second, the obtained $L^{off}$ is interpolated in real time to obtain L(ϑ) by exploiting relation (9).

To formulate LMIs for the discretized system, the continuous fuzzy model Equation (9) can be discretized using Euler approximation with sampling time $\Delta t_{s}$:

(23) $$\begin{array}{c} x\left(k+1\right)=x\left(k\right)+\sum_{i=1}^{32}h_{i}\left(\vartheta \left(t\right)\right)\left\{A_{i}x\left(k\right)+B_{i}u\left(k\right)\right\}\Delta t_{s} \end{array}$$

(24) $$\begin{array}{c} \;\;\;\;=\sum_{i=1}^{32}h_{i}\left(\vartheta \left(t\right)\right)\left\{\underset{A_{di}}{\underset{︸}{\left(I+A_{i}\Delta t_{s}\right)}}x\left(k\right)+\underset{B_{di}}{\underset{︸}{\left(B_{i}\Delta t_{s}\right)}}u\left(k\right)\right\} \end{array}$$

The LQR optimal control objective is to find a state feedback control u(k)=−Kx(k), where *K* is the gain for the system matrix, that minimizes the following objective function:

(25) $$\begin{array}{c} J=\sum_{k=0}^{\infty }\left[x^{T}\left(k\right)Qx\left(k\right)+u^{T}\left(k\right)Ru\left(k\right)\right] \end{array}$$

Introducing a Lyapunov function for each vertex of the polytopic model:

(26) $$\begin{array}{c} V\left(x\left(k\right)\right)=x^{T}\left(k\right)Px\left(k\right) \end{array}$$

that satisfies the following conditions:

(27) $$V(x_{0})<\gamma$$

(28) $$V\left(x\left(k+1\right)\right)-V\left(x\left(k\right)\right)+x^{T}\left(k\right)Qx\left(k\right)+u^{T}\left(k\right)Ru\left(k\right)<0$$

Integrating the Equation (28) and substituting u(k)=−Kx(k) yields

(29) $$\begin{array}{c} \sum_{k=0}^{\infty }\left[x^{T}\left(k\right)Qx\left(k\right)+u^{T}\left(k\right)Ru\left(k\right)\right]<V\left(x_{0}\right)=x_{0}^{T}Px_{0}<\gamma \end{array}$$

The above equation ensures that LQR objective function is bounded by the optimal value γ for all the polytopes. The increment of Lyapunov function (26):

(30) $$\Delta V\left(k+1\right)=x^{T}\left(k\right)A_{d}{\left(\vartheta \right)}^{T}PA_{d}\left(\vartheta \right)x\left(k\right)-x^{T}\left(k\right)Px\left(k\right)$$

For the closed-loop system the Lyapunov function becomes

(31) $$\Delta V\left(k+1\right)=x^{T}\left(k\right){\left(A_{d}-B_{d}K\right)}^{T}P\left(A_{d}-B_{d}K\right)x\left(k\right)-x^{T}\left(k\right)Px\left(k\right)$$

The previous inequality Equations (29) and (31) can be rewritten as follows:

(32) $$x_{0}^{T}Px_{0}<\gamma$$

(33) $${\left(A_{d}-B_{d}K\right)}^{T}P\left(A_{d}-B_{d}K\right)-P+Q+K^{T}RK<0$$

Applying duality principle $A_{d}\to A_{d}^{T},B_{d}\to C_{d}^{T},K\to L^{T}$ [19] to LQR inequality Equation (33) results:

(34) $${\left(A_{d}^{T}-C_{d}^{T}L^{T}\right)}^{T}P\left(A_{d}^{T}-C_{d}^{T}L^{T}\right)-P+Q+LRL^{T}<0$$

By applying some algebraic manipulations to Equations (32) and (34), some changes of variables $\left(Q=H^{T}H,Y=P^{-1}\right)$ and Schur complement lead to the following LMIs optimization:

(35) $$\begin{array}{c} \left[\begin{array}{cc} \gamma I & I\\ I & Y \end{array}\right]>0\\ \left[\begin{array}{cccc} -Y & YA_{d}-W^{T}C_{d} & YH^{T} & W^{T}\\ A_{d}^{T}Y-C_{d}^{T}W & -Y & 0 & 0\\ HY & 0 & -I & 0\\ W & 0 & 0 & -R^{-1} \end{array}\right]<0 \end{array}$$

The optimal gain can be found by $L={\left(WY^{-1}\right)}^{T}$. The solution involves optimization at each time step, due to the varying system matrices ($A_{d},B_{d}$), which are computationally expensive and slow. Instead, we exploit the method developed in Section 2.1.1. Any nonlinear system can be represented in the form (24), which means the offline gain will be found at the system vertices (polytopes) of these systems and later on interpolated online using fuzzy membership grade function (11) for the TS model (5). Therefore, for the 5 scheduling variables, 32 system matrices can be obtained at the polytopes, resulting in 32 LMIs and offline gain. The gain obtained from solving LMIs will be called $L_{i}^{off}\;\in \left[1,\cdot \cdot \cdot ,32\right]$. All the steps for obtaining the LMIs are mentioned in Algorithm 1.

### 4.2. Disturbance and Noise Matrix

The disturbance of the vehicle model and sensor noise is modeled by Gaussian distribution whose mean is set to zero, and covariance is obtained experimentally. The disturbance and the noise of the model are found experimentally, and some of the sensor noise from the manufacturer’s data sheet. The scan matching covariance is obtained via a sampling-based covariance estimate. However, a second method based on the Hessian matrix can also be used [20]. The disturbance in the model is considered to be higher than the measurement noise to compensate for uncaptured dynamics during parameter estimation. The following covariances ($m^{2}$) for the experiment were set for the disturbance (Q) and noise (R) matrices, respectively:

(36) $$\begin{array}{c} Q=\mathrm{diag}(0.15,0.05,0.15,0.25,0.25,0.1) \end{array}$$

(37) $$\begin{array}{c} R=\mathrm{diag}(0.04,0.0187,0.0225,0.0225,0.01) \end{array}$$

### 4.3. Switching Estimator Design

During the experiment phase, the scheduling variable θ:[−π,0] does not yield a better approximation of the nonlinear function. This can be seen by substituting the values in element $A_{41},A_{42},A_{51},\;\mathrm{and}\;A_{52}$ in Equation (5) and taking only these subelements:

(38) $$\begin{array}{c} A_{4:5,1:2}\left(\theta \right)=\left[\begin{array}{cc} \cos(\theta ) & -\sin(\theta )\\ \sin(\theta ) & \cos(\theta ) \end{array}\right] \end{array}$$

(39) $$A_{4:5,1:2}\left(-\pi \right)=\left[\begin{array}{cc} -1 & 0\\ 0 & -1 \end{array}\right],\;A_{4:5,1:2}\left(0\right)=\left[\begin{array}{cc} 1 & 0\\ 0 & 1 \end{array}\right]$$

The above polytopic systems are true considering the minimum and maximum vertices in cos function, but it is not valid for sin function. To accurately model this effect, the θ scheduling variable is chosen for each quadrant $\vartheta_{4j}=\left[0,\frac{\pi }{2},-\pi ,\frac{-\pi }{2}\right],\;j\in \left[1,..,4\right]$. This improvement provides the system vertex to reach all the possible values:

(40) $$A_{4:5,1:2}\left(0\right)=\left[\begin{array}{cc} 1 & 0\\ 0 & 1 \end{array}\right],\;A_{4:5,1:2}\left(\frac{\pi }{2}\right)=\left[\begin{array}{cc} 0 & 1\\ 1 & 0 \end{array}\right]$$

(41) $$A_{4:5,1:2}\left(-\pi \right)=\left[\begin{array}{cc} -1 & 0\\ 0 & -1 \end{array}\right],\;A_{4:5,1:2}\left(\frac{-\pi }{2}\right)=\left[\begin{array}{cc} 0 & -1\\ -1 & 0 \end{array}\right]$$

**Figure 5:**
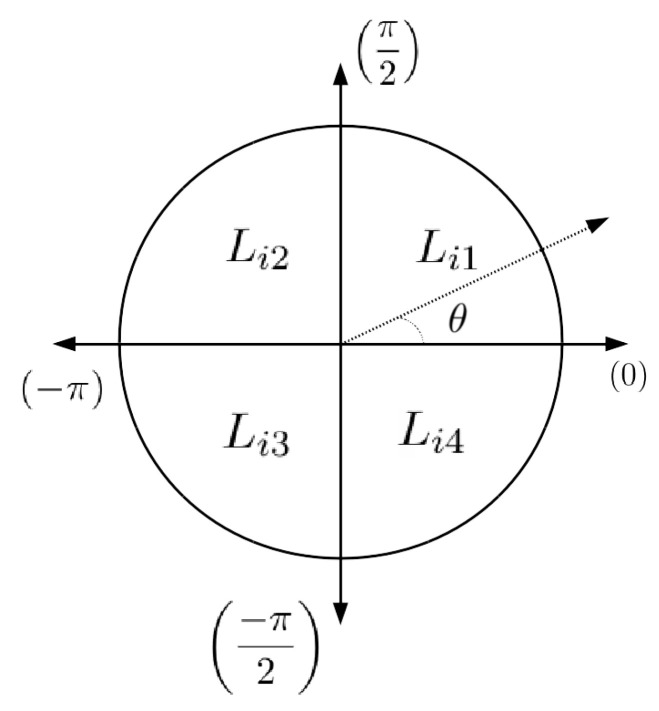
A defined sector that is designed to capture kinematic system matrices to obtain offline gain. Later on, using the same sector approach, online interpolated gains are obtained.

**Figure 6:**
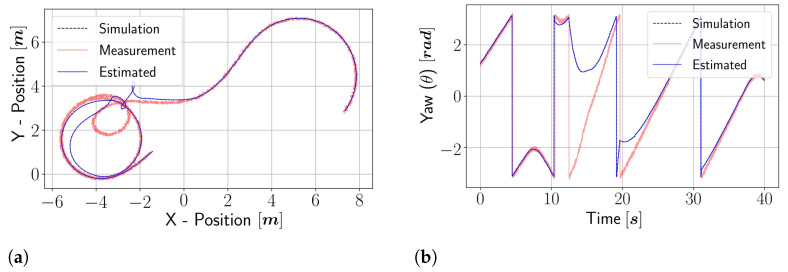

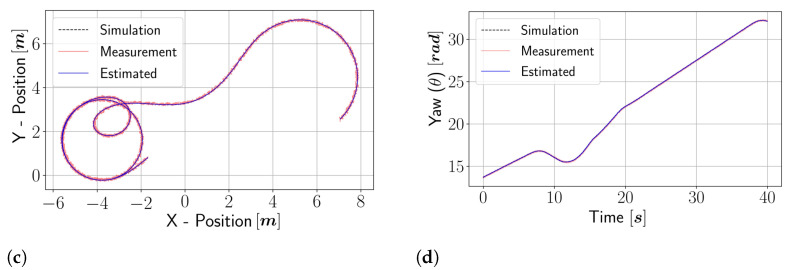
Estimation comparison between wrapped yaw measurement and unwrap measurement. Panels (**a**,**b**) represent the position of vehicle and yaw measurement in the global frame, respectively, both of these states are estimated using yaw measurement between [−π,π]. Panels (**c**,**d**) represent the position of vehicle and yaw measurement in the global frame, respectively, both of these states are estimated using continuous yaw measurement. (**a**) X–Y global position on yaw measurement between [−π,π]. (**b**) Yaw sensor measurement. (**c**) X–Y global position on yaw measurement between [−∞,∞]. (**d**) Yaw transformed to continuous measurement.

For the implementation of this system, every four sections of the quadrant are considered and the LMIs for all the quadrants are computed offline, following the steps mentioned in Algorithm 1.   
**Algorithm 1:** Offline Gain Optimization Algorithm1.Define maximum and minimum scheduling variables for four sectors:
(a)$\left[\left[{\underline{\vartheta }}_{1},{\bar{\vartheta }}_{1}\right],\left[{\underline{\vartheta }}_{2},{\bar{\vartheta }}_{2}\right],\left[{\underline{\vartheta }}_{3},\bar{\vartheta_{3}}\right],\left[{\underline{\vartheta }}_{41},{\bar{\vartheta }}_{41}\right],\left[{\underline{\vartheta }}_{5},{\bar{\vartheta }}_{5}\right]\right]$(b)$\left[\left[{\underline{\vartheta }}_{1},{\bar{\vartheta }}_{1}\right],\left[{\underline{\vartheta }}_{2},{\bar{\vartheta }}_{2}\right],\left[{\underline{\vartheta }}_{3},\bar{\vartheta_{3}}\right],\left[{\underline{\vartheta }}_{42},{\bar{\vartheta }}_{42}\right],\left[{\underline{\vartheta }}_{5},{\bar{\vartheta }}_{5}\right]\right]$(c)$\left[\left[{\underline{\vartheta }}_{1},{\bar{\vartheta }}_{1}\right],\left[{\underline{\vartheta }}_{2},{\bar{\vartheta }}_{2}\right],\left[{\underline{\vartheta }}_{3},\bar{\vartheta_{3}}\right],\left[{\underline{\vartheta }}_{43},{\bar{\vartheta }}_{43}\right],\left[{\underline{\vartheta }}_{5},{\bar{\vartheta }}_{5}\right]\right]$(d)$\left[\left[{\underline{\vartheta }}_{1},{\bar{\vartheta }}_{1}\right],\left[{\underline{\vartheta }}_{2},{\bar{\vartheta }}_{2}\right],\left[{\underline{\vartheta }}_{3},\bar{\vartheta_{3}}\right],\left[{\underline{\vartheta }}_{44},{\bar{\vartheta }}_{44}\right],\left[{\underline{\vartheta }}_{5},{\bar{\vartheta }}_{5}\right]\right]$
2$A_{ij},B_{ij}\;i\in \left[1,2,\cdots ,32\right],j\in \left[1,2,\cdots ,4\right]\leftarrow$ Using Equation (5) obtain 32 polytopic systems for each scheduling variables (a)–(d);3.$A_{dij},B_{dij}\leftarrow$ Discretize using sampling time (Δt);4.$L_{ij}^{off}\leftarrow$ Optimize formulated LMIs Equation (35);

The estimator gains $L_{ij}^{off},\;j\in \left[1,..,4\right]$ are obtained for each quadrant, and then particular gains are applied according to the region in which the previous yaw estimate (${\hat{\theta }}_{wrap}$) lies. For example, in Figure 5, θ lies in the first quadrant, then $L_{i1}^{off}$ gain will be weighted using the TS fuzzy interpolation function depending on the scheduling variable $\left[0,\frac{\pi }{2}\right]$ to obtain the current online gain. Likewise, for each quadrant, the $L_{ij}^{off}$ is interpolated for the current states using the membership function. Algorithm 2 presents the method to obtain the fuzzy gain interpolation scheme.
**Algorithm 2:** Gain Interpolation AlgorithmL_interpolation$\left(\vartheta \left(k\right),L_{ij}^{off}\right)$;**Input**: Scheduling variable ϑ(k)**Output**: Interpolated gain *L*$\bar{\vartheta_{i}},\underline{\vartheta_{i}}\leftarrow \mathrm{Load}\;\mathrm{scheduling}\;\mathrm{variables}$;$\mu_{i1}\left(k\right)$, $\mu_{i2}\left(k\right)\leftarrow$ Compute fuzzy sets for all the ϑ(k) using Equations (7) and (8);$h_{i}\left(\vartheta \left(k\right)\right)\leftarrow$ Compute fuzzy interpolation function using Equation (11);$L\leftarrow \sum_{i=1}^{32}h_{i}\left(\vartheta \left(k\right)\right)L_{ij}^{off}$;

   The algorithm for the proposed state estimation technique can be referred from Algorithm 3. The wrap() function is used to discretize the θ value between [−π,π], and the unwrap() function is used to change the θ value to continuous value. This is required as the yaw measurement from the sensor provides a measurement between [−π,π], which is discontinuous. The measurement discontinuity results in wrong estimates due to the lagging or leading effect of the measurement. In Figure 6, the wrong estimation of states is depicted. Due to the abrupt change in yaw measurement in Figure 6b, the robot position is wrongly estimated in Figure 6a. It can be seen in Figure 6b that at around 12.5 s, the estimated yaw slightly leads the measurement, due to which the estimator tries to track the measurement data, which results in the full turn of the robot at location [−1.8,4]. The same phenomenon at 19 s also occurs when the estimated yaw lags behind the measurement data. Changing the discontinuity using the unwrap() function provides an accurate estimation of all the states, which is shown in Figure 6c,d.

**Algorithm 3:** State Estimation Algorithm

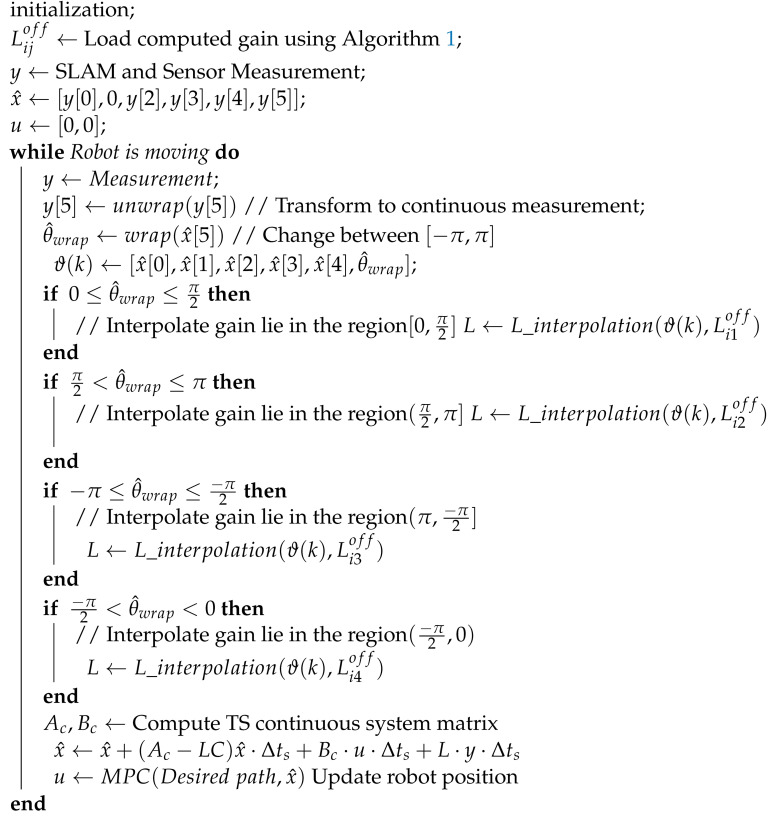



## 5. Results

**Figure 7:**
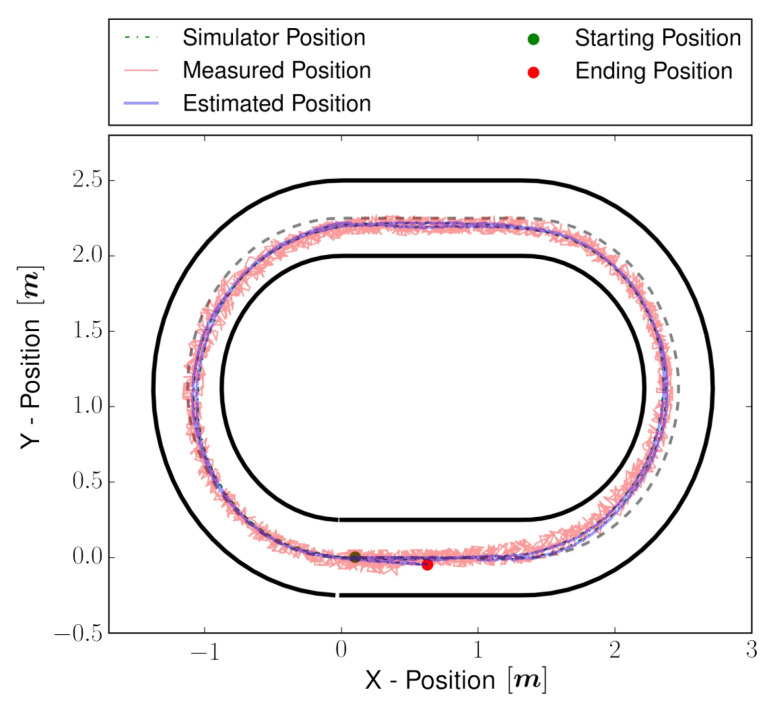
Vehicle estimated trajectory in the presence of sensor disturbance during the simulation. As per the controller policy, the vehicle tried to follow two laps of the center-line track.

To validate the estimator performance, experiments were performed in the simulator as well in a real environment. First, the estimator is tested using manual control input, and once it is proved to be working, an NMPC controller is utilized to follow a track. The vehicle is 41 cm long and 21 cm wide, and the track is 50 cm in width. The setting of the controller is tuned to complete two laps while achieving a longitudinal velocity of 0.8 m/s, keeping the vehicle inside the track and closer to the center track, shown as a dotted line in Figure 7. The objective of the validation is to (i) estimate full states correctly, including unmeasured state $v_{y}$, in the simulator as well as real vehicle, and (ii) validate real-time performance. For the simulator, the NRMSE evaluation metric is used to compare the error between the simulated and estimated state, which can be computed by:

(42) $$\mathrm{NRMSE}=\frac{\sqrt{\sum_{i=1}^{N}{\left(x-\hat{x}\right)}^{2}}}{x_{\max}-x_{\min}}$$

### 5.1. Simulation

The vehicle simulator is developed using the vehicle full state dynamics obtained in Section 2.1. The perturbations and noises are injected into the vehicle model and sensor model, respectively, to simulate the real world. The perturbation and noises are kept a little higher than the actual one to ensure the estimator works even in the worst cases. By analyzing Figure 7, the estimated position is very close to the simulator state of the vehicle. The NRMSE error for all the estimated states is presented in Table 5.

**Figure 8:**
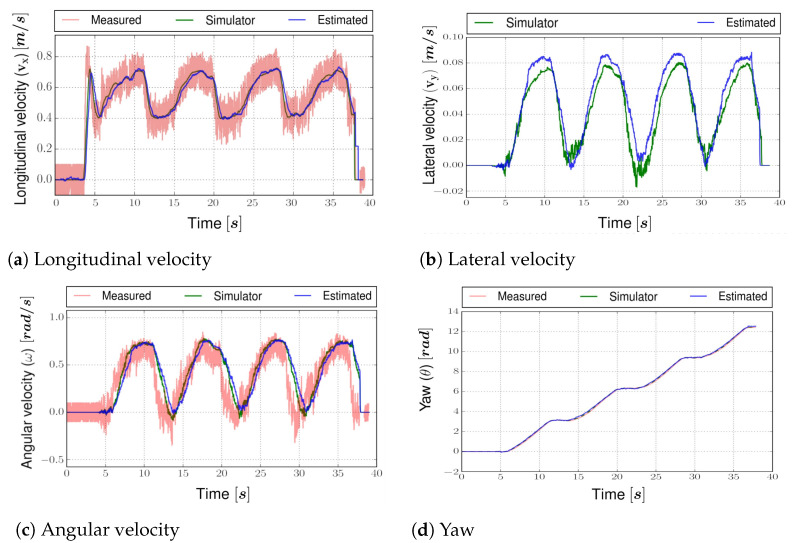
Estimated states on vehicle simulator for two laps.

The rest of the estimated states are compared in Figure 8. The lateral velocity ($v_{y}$), which cannot be measured directly, has an *NRMSE* value of 0.0323 (Figure 8b). The errors of all the dynamic estimated states are within a certain range, which indicates that the estimator is fully working and ready to be tested on the real vehicle.

### 5.2. Real Experiment

Note that the *RMSE* error is not used here due to a lack of ground truth measurement. The validation for this experiment is performed visually.

**Figure 9:**
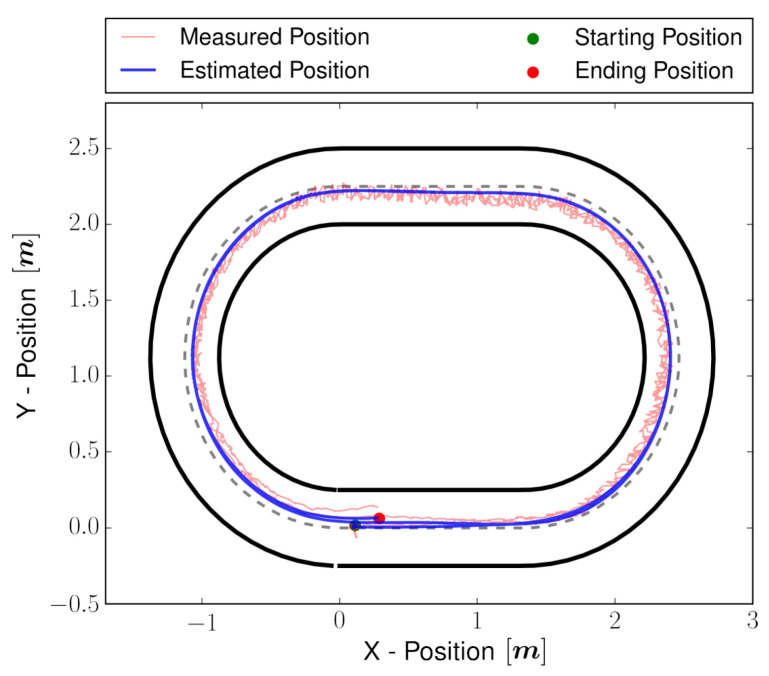
Vehicle estimated trajectory in the presence of sensor disturbance during the real experiment. As per the controller policy, the vehicle tried to follow two laps of the center-line track.

**Figure 10:**
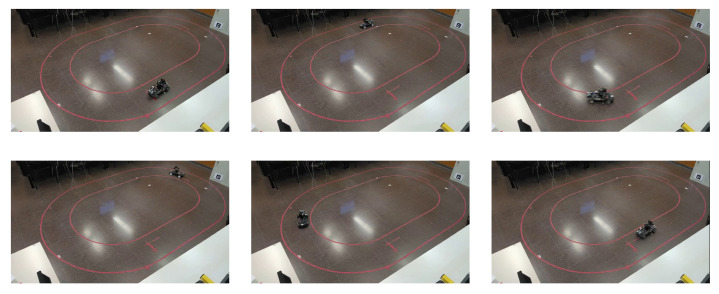
Snapshots of the vehicle following the center line of the oval track. The vehicle motion is represented by a sequence of images clockwise from the upper-left to the bottom-left.

The estimated X–Y position for the real experiment is shown in Figure 9. Some snapshots for visual validation are shown in Figure 10 and compared to Figure 9; it can be noticed that the estimated position of the vehicle matches the real position of the vehicle in the snapshots. For properly substantiated illustration, the media attached (https://youtu.be/Oey2ZxsxlnY (accessed on 29 March 2022)) shows the estimated states and NMPC controller performance. The media validates the performance of the estimator design.

**Figure 11:**
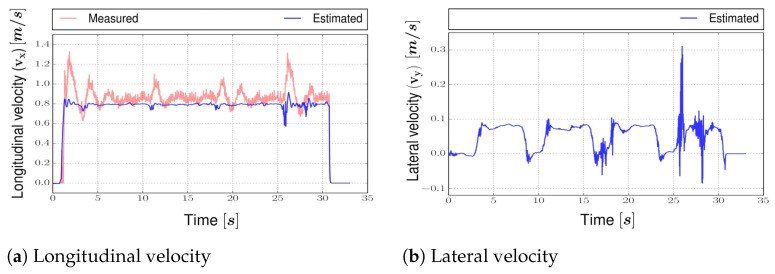

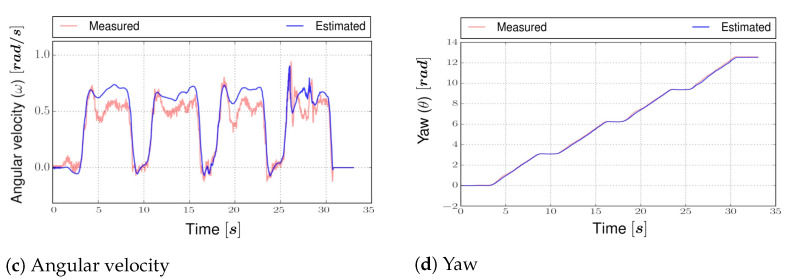
Estimated states on real vehicle for two laps.

Additional estimated states are shown in Figure 11. The velocity obtained from the motor encoder is very noisy and inaccurate (see Figure 11a), but the TS Kalman filter can provide quite a clean estimation. Similarly, this happens with the angular velocity. The lateral velocity was not measured and is successfully estimated.

**Figure 12:**
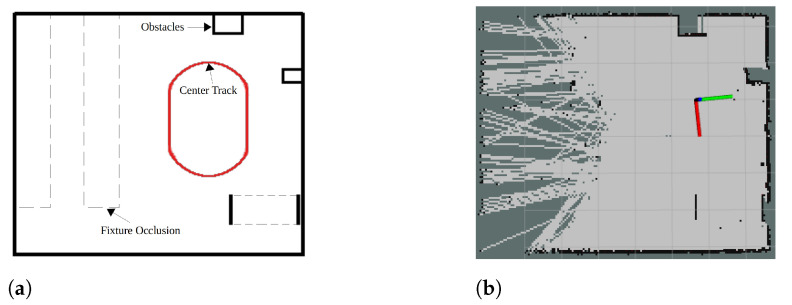
Comparison of ground truth and final map obtained. (**a**) The ground truth image represents the environment where the testing is performed. (**b**) The final map formed after completion of two laps.

The final corrected map after completing the laps is shown in Figure 12b. Figure 12a represents the environment which includes fixtures, obstacles, and free space. There are some unoccupied cells, particularly when the LIDAR scan is hindered by the fixtures present in the environment. For this reason, the error between ground truth and map is only calculated for properly scanned areas, i.e., the fixture occlusion region is excluded (see Figure 12a). The intersection-over-union (IOU) score of 0.9388 is obtained between the ground truth and the corrected map.

### 5.3. Simulated vs. Real Experiment

It is worth noting that the time taken for completing two laps in the simulator and real experiments are 38 s and 32 s, respectively.

The real experiment completed the lap faster than the simulation due to strictly maintaining a longitudinal velocity of 0.8 m/s. There are discrepancies between some estimated states which are due to different control input in simulation and real experiment phase or mismatch of the simulator model from the real vehicle model. Table 6 shows the minimum and the maximum absolute differences between estimated and measured states, as well as their standard deviations for both the simulator and real platform experiment. The minimum deviations for the real experiment are similar to those of the simulator, while there is a deviation of about 10 cm for real experiment values compared with simulator values; this is because the standard deviation of simulation measurements is set higher than the real standard deviation. This can also be validated from the standard deviation values in the simulator and real experiments. Even though the performance of the estimator in the real experiment cannot be verified due to the lack of ground truth, the values in the real experiment appear to be similar to those in the simulator, suggesting that the estimator performs similarly.

## 6. Conclusions

This paper has presented an approach for full-state estimation of an autonomous vehicle using a TS Kalman filter as well as imprecise scan matching and measurements. Further, this technique can also correct the map obtained from the noisy LIDAR scan end-points matching. The proposed approach provides a stable and optimal solution in the presence of model disturbance and measurement noises at a high update rate of 100 Hz for state estimation, and simultaneous correction of the obtained map. The update rate can go much faster than 100 Hz; the only limitation is the update rate of the sensor measurements. The computational cost of the proposed approach is fairly low if compared with EKF SLAM, which depends on the number of landmarks. The result produced motivates the usage of the TS-based state estimation and mapping in the field of autonomous vehicles and robotics.

The proposed estimation technique depends on the model of the system and the measurement model with certain disturbance and noise, respectively. This requirement is sometimes hard to fulfill, and also, for some systems, the parameters change within a certain range, for example, the tire coefficient of the vehicle. For such a kind of system, online model learning techniques need to be incorporated [21,22]. The proposed method can also be scaled to a high-DoF system if the system model is known, and extra sensors can be installed, for example, a height sensor, such as a barometer, or range sensor can be used to detect z-pose. The proposed scan matching technique can be applied to an unknown environment but the accuracy may degrade in a highly dynamic environment. For such cases, robust scan matching techniques need to be applied, such as in [23].

As future work, the proposed approach will be benchmarked against competing approaches.

## Figures and Tables

**Table 1:** The list of symbols used in this article.

Symbol	Description
$v_{x}$	Longitudinal velocity of vehicle in center of gravity (CoG) frame (C), see Figure 2.
$v_{y}$	Lateral velocity of the vehicle in CoG frame (C).
ω	Angular velocity of the vehicle in CoG frame (C).
*X*	Global position of the vehicle in *X*-axis frame (O).
*Y*	Global position of the vehicle in *Y*-axis frame (O).
θ	Orientation of the vehicle with respect to the *x*-axis of the frame (O).
*D*	Duty Cycle of motor, normalized between [0,1].
δ	Steering angle.

**Table 2:** Vehicle model parameters.

Parameters	Values	Description
*m*	2.424 kg	Mass of the vehicle
$l_{f}$	0.1377 m	Distance from CoG to front wheel
$l_{r}$	0.1203 m	Distance from CoG to rear wheel
ρ	1.225 kg/m${}^{3}$	Air density
$C_{m0}$	9.4685 N	Motor parameter 1
$C_{m1}$	0.6672 kg/s	Motor parameter 2
$C_{0}$	2.6104 kg/s	Resistive driveline parameter
$C_{1}$	−0.00213 N	Static friction force
$C_{D}A$	0.466 m${}^{2}$	Coefficient of drag multiplied with area
$C_{af}$	1.2354 N/rad	Front wheel cornering stiffness
$C_{ar}$	1.4532 N/rad	Rear wheel cornering stiffness
$I_{z}$	0.02 kg·m${}^{2}$	Moment of inertia

**Table 3:** Scheduling variables, i.e., states or inputs on which the system matrix A(ϑ) and B(ϑ) are dependent. The maximum and minimum value of these variables can define any polytopic system.

Variables $\left(\vartheta_{i}\left(t\right)\right)$	$\min\left(\vartheta_{i}\left(t\right)\right)=\underline{\vartheta_{i}}$	$\max\left(\vartheta_{i}\left(t\right)\right)=\bar{\vartheta_{i}}$
** $v_{x}$ **	−5.0	5.0
** $v_{y}$ **	−3.0	3.0
** ω **	−1.5	1.5
** θ **	−π	π
** δ **	−0.35	0.35

**Table 4:** Noise of dedicated sensors or algorithms used in the experimental platform to measure or estimate the rough information.

States	Sensors	Covariance $\left(\sigma^{2}\right)$ [m${}^{2}$]
** $v_{x}$ **	Motor encoder	0.04
** ω **	IMU	0.0187
***X***	Scan matching	0.0225
***Y***	Scan matching	0.0225
** θ **	IMU	0.01

**Table 5:** NRMSE evaluation of estimated states.

States	$v_{x}$	$v_{y}$	ω	*X*	*Y*	θ
NRMSE	0.0781	0.0323	0.0913	0.0157	0.0175	0.0198

**Table 6:** Evaluation between estimated states and measured states for the experiment in the simulator and real platform. The min and max header represent the minimum and maximum absolute difference between estimated and measured states, respectively. The std header represents the standard deviation between estimated and measured states.

States	min [m]		max [m]		std [m]	
	Sim	Real	Sim	Real	Sim	Real
X	0.124	0.094	0.405	0.317	0.159	0.116
Y	0.137	0.119	0.344	0.241	0.138	0.126

## Data Availability

All the required data is included in the paper.
